# Use of a structure-borne sound-based in-process sensor system to identify Weld seam irregularities during electron beam welding

**DOI:** 10.1038/s41598-024-73797-8

**Published:** 2024-09-27

**Authors:** Christian Wolf, Niklas Sommer, Stefan Böhm

**Affiliations:** Department for Cutting and Joining Manufacturing Processes - Institute for Production Technologies and Logistics, University of Kassel, Kurt-Wolters-Straße 3, 34125 Kassel, Germany

**Keywords:** Electron beam welding, Structure-borne sound emission, In-process monitoring, Weld seam irregularities, Machine learning models, Engineering, Mechanical engineering

## Abstract

In this publication, an in-process quality assurance method for electron beam welding based on a structure-borne sound emission test for the detection of weld irregularities arising in the process is presented. For this purpose, different sheet materials, i.e., AISI 304, AZ31 and AlMg3, were welded in a butt-joint and the resulting process noises were recorded by means of two acoustic emission sensors specifically designed for structure-borne sound. During the welding experiments, typical irregularities, e.g. incidence points, pore lines and cracks, were deliberately induced. Subsequently, the recorded acoustic signals were examined with regard to defect-specific abnormalities. Various methods in the time and frequency domain as well as pre-trained machine learning models were used to analyze the acoustic emission data. The results show that the investigated irregularities can be identified and distinguished from other process emissions, eventually enabling a robust means of identification for weld seam irregularities and, thus, opening pathways towards cost-effective in-process quality control.

## Introduction

Owing to the comparatively high achievable weld seam quality, i.e., small fusion zones, minimal welding distortion, and, thus, the possibility to fabricate components near-net-shape, electron beam welding (EBW) is often used within the last manufacturing steps of a production line^1^, exemplary in the aerospace or automotive sector^2–5^. However, the use of fully-automated production lines without adequate quality detection capabilities can lead to serious production losses as a result of undetected process fluctuations. Consequently, users of EBW technology demand excellent reproducibility through maximum process stability, both from an economic and security point of view^1,4^. At the same time, the demands on process stability and reproducibility grow with increasingly complex joining tasks and, thus, require ever more constant boundary conditions.

Despite the fact that in EBW all process parameters such as beam current, lens current, acceleration voltage, etc. are available as electrical quantities which can be easily measured and recorded, it is virtually impossible to derive the weld quality from recording these parameters. In addition to insufficient demagnetization of ferromagnetic materials, e.g., steels, leading to impermissible beam deflection^6^, cathode-induced weld seam irregularities^7,8^ can lead to unwanted and currently undetectable deviations in weld seam quality. Errors in the preparation of abutting edges as well as component- or material-related process influences leading to pores or cracks can also not or only insufficiently be detected through parameter monitoring during EBW^9–12,5^. The obvious lack of detectability means that the inspection effort in the downstream production line must be significantly increased. This increased need for inspection is inevitably accompanied by either rising cost per component and/or lower productivity to allow for thorough inspection. Also, the demand for a complete documentation of the EBW process including quality inspection can so far only be realized via downstream inspection methods, clearly associable with the above-mentioned consequences for costs and productivity. For these reasons, there is substantial demand for a reliable in-line inspection system during EBW.

A promising approach to assess the weld quality during the process, i.e., in operando, is the analysis of emitted process noise during the welding process, as has been documented e.g. by Steffens et al.^13^ and Wolf et al.^14^. Yet, due to the vacuum conditions in the working chamber of an EBW system, insufficient propagation of airborne sound renders the detection of irregularities impossible. However, process-inherent, structure-borne sound signals may be utilized and have proved to enable the detection of metallurgically induced defects such as cracks, pore lines or incidence points^15,16^. Due to the high sensitivity of structure-borne sound transducers, process monitoring of very small melt pools, i.e., the ones typically found in EBW, is also possible^14^.

## State of the art

In the following, an overview of the state of the art and the current findings and problems that arise in the process monitoring of EBW are described.

Inline inspections of welding processes have been state of the art for years. With various arc welding processes, a direct correlation to the process (e.g. short circuit, droplet separation) can be established via the electrical currents and voltages^17^. Optical methods using cameras or similar can also detect certain abnormalities in the arc or the melt comparatively easy due to the relatively large welding zone^18,19^. However, the inline testing of beam welding processes is much more difficult because the electrical parameters in the beam generator only allow for an indirect correlation to the process quality. Furthermore, the resulting melt pools can be considerably smaller and deeper^2,5,10^, the energy input is more focused^5^ and the cooling rates are significantly higher^2,20–22^. The monitoring of a beam welding processes is therefore usually accompanied by the recording of process emissions, which emanate from the process itself^23^. Moreover, typical methods of downstream non-destructive testing, such as radiographic testing, cannot usually be carried out during a welding process in an industrial setup^24^.

In principle, welding processes emit various emissions in the form of sound waves, electromagnetic waves and particles, of which both the acoustic and electromagnetic emissions can be used to detect process events^5,23,25^.

Furthermore, the imaging of the workpiece surface by means of backscattered electrons, often called electron optical insight or ELO monitoring, can be used to detect superficial cracks^26^ or other inhomogeneities. The signal from a built-in CCD camera can also be used to detect weld defects, e.g., cracks, although the contrast and resolution of such camera images are often inferior to the electron-optical image. In addition, these processes may not be considered true in-line processes, except for the monitoring of the system parameters, as the detection of superficial defects requires an image of the seam surface past the actual process. An exception to this is the so-called “ELO-Online”, in which the electron beam leaves the weld for a few milliseconds by means of rapid beam deflection in order to record an image of the weld with its surroundings^27^. Even though the electron beam only leaves the weld for a very short period of time compared to the welding time for imaging, a feedback effect on the process cannot be ruled out.

Another possibility is to use the resulting X-rays for process monitoring. Similar to the backscattered electrons, conclusions about the focus position in the process or the position of the beam relative to the weld joint can be determined by evaluating the radiation intensity^28^.

Further weld seam irregularities during the electron beam process cannot yet be adequately detected according to the current state of research, therefore clearly limiting the evaluation of the component quality. While it is possible to log the focus current and, thus, indirectly detect a correct focus position, a large number of other irregularities, such as pores, cracks or binding defects, cannot be directly detected based on machine input parameters alone. Optical methods that evaluate the infrared radiation of the process and the melt can identify pores or blowholes, since a short-lived signal drop must be taken into account when these occur^29^. As metal vapor is generated during the welding process, however, optical process monitoring systems have not proven to be effective in the long term because the metal vapor may deposit on optical lenses, which significantly impairs the signal quality with time and, thus, makes consistent measurements problematic^29^.

Acoustic emission analysis has been used in various scientific publications to monitor the EBW process. Due to the vacuum in the working chamber of an EBW system, there is no propagation of airborne sound, which is why structure-borne sound was always recorded and evaluated. The first investigations were carried out by Steffens and Crostack as early as 1973, with a particular focus on the frequencies occurring and their dependence on the propagation in the metal^13^. Here, the structure-borne sound signal was used in the sense of a time-of-flight measurement to obtain an indication about the position of the sound source. The first applications in EBW took place in 1975^30^, where four types of structure-borne noise signals were distinguished: (i) noise from the welding process (phase transformation, plastic deformation and crack formation), (ii) noise from the equipment (e.g. drives), (iii) combinatory noise due to the interaction of workpiece and equipment (e.g. friction or temperature effects due to thermal expansion) and (iv) noise from the environment, which the authors suppressed by means of a high-pass filter at 50 Hz^31^. An assignment of the structure-borne sound signal to individual causes, i.e., welding irregularities, was not carried out due to the lack of evaluation possibilities^31^. At about the same time, acoustic emission analyses were carried out in the Soviet Union, in which cracking could be detected during and after the welding process^15,16^.

All structure-borne sound analyses from the 1970s have in common that only time signals and amplitudes were evaluated. The possibility to perform a frequency analysis in real time during the process was not possible due to an apparent lack of computational hard- and software. As a result, signal-irregularity-assignments were only possible in a very unspecific manner.

In 1998, Crostack et al. succeeded in using improved structure-borne sound sensors and newer recording technology to localize the point of origin of sound events - in this case cracking in the weld seam - with a high degree of accuracy and without any knowledge of the sound velocity of the welding materials^32,33^. However, the measured sound signals could not be assigned to any other defect categories.

More recent investigations in the field confirm the above findings^34^. Here, unambiguous statements could be made about the welding depth, i.e., penetration yes/no, and the crack formation during EBW of Ti6Al4V, whereby only the amplitude signals were evaluated. The irregularities themselves were not detected *during* the welding phase, but only those irregularities that occurred after the process during the solidification phase^15^. Evidently, these findings were to be expected on basis of the simultaneous nature of melt pool dynamics, shrinkage as well as residual stress/distortion development.

While investigations carried out in the 70s and 80s of the 20th century showed a feasibility in principle, the evaluation of obtained signals was challenging due to missing automation routines. Yet, recent research works have definitely shown that an improved evaluation is possible using computer-aided analyses. According to the current state of research, acoustic emission analysis or acoustic process monitoring can be regarded as a suitable method for process monitoring, as the emitted process noises can be recorded and, simultaneously, information can be extracted from the signal at the same time. The characteristics of the latter can then be used to find irregularities. However, a definitive correlation between weld seam irregularities and corresponding sound events has not yet been established in the context of EBW. Statements from the sound data as to which defects are involved and whether a defective weld seam is present due to the defect size, type and location have not yet been made. This fundamental lack of understanding shall be overcome in the present investigation which utilizes computer-aided data analyses and machine learning approaches to correlate sound events to defect formation during EBW.

## Results

### Reproducible inducing of Weld seam irregularities

**Fig. 1:**
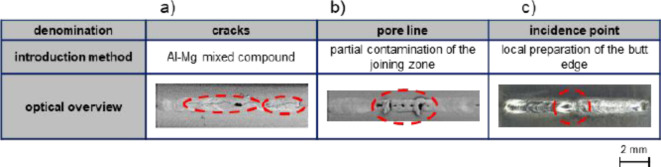
Denomination and representative overviews of deliberately introduced fault types as well as methods for introduction. (**a**) Cracks, (**b**) pore line and (**c**) incidence point.

As the study of literature has shown, an in-line sound emission system should be able to detect relevant irregularities of electron beam manufacturing processes. However, in order to identify the defect-specific sound signatures, a robust and reproducible means to deliberately induce relevant weld seam irregularities is necessary. Figure 1 illustrates the three investigated fault types as well as their introduction method and surficial appearance. For the fault type crack (cf. Figure 1a), for example, a dissimilar joint of an Al- and a Mg-alloy was used, which has a high susceptibility to cold cracking due to the formation of intermetallic phases^35,36^. As the corresponding ELO-image visualizes, the weld is characterized by a pronounced crack along the welding direction. The fault type pore line (cf. Figure 1b) was created by a partial preparation of the abutting edges in such way that the oxide layer stemming from the electric discharge machining process used to extract the specimens was not removed in the center of the joint. Clearly, the induced contamination leads to a substantial pore formation in the center of the weld seam, as the ELO-image shows. Fault type incidence point (cf. Fig. 1c) was created by a mechanical preparation of the butt-edge on the backside by creating material through a triangular 45° phase. The colored rectangle on the macroscopy image clearly indicates the formation of an incidence point along the welding trajectory, while the surrounding weld is characterized by a homogeneous bead formation.

In order to be able to assign the occurring changes in the sound signals to specific fault types, it was first important to generate the individual fault types in a reproducible manner. As the provided overview images underline, it was possible to introduce the defects with a high degree of accuracy and the intended macroscopic effects.

### Detection of Weld seam irregularities by acoustic emission

**Fig. 2:**
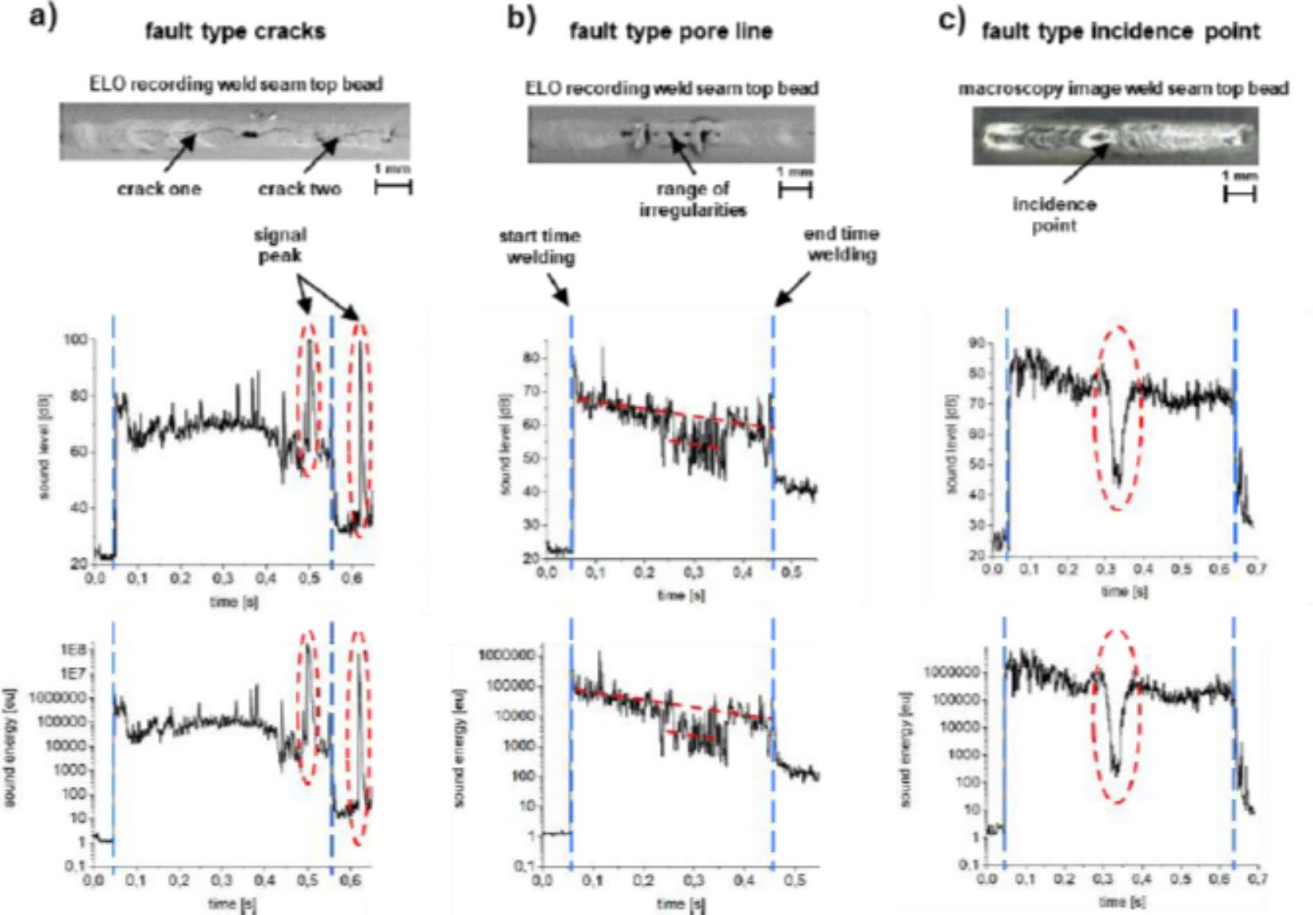
Comparison of the different fault types (upper row) with evaluation of the sound level (middle row) and the sound energy (bottom row) in the time domain for (**a**) fault type crack (**b**) fault type pore line and (**c**) fault type incidence point. Welding direction was left to right.

Figure 2 shows representative acoustic emission data obtained for the aforementioned fault types crack, pore line and incident point together with the exemplary overview images. For the fault type crack (cf. Figure 2a), two cracks occurred along the weld seam. In the corresponding sound level recordings, two distinct sound peaks can be seen in the signal. In terms of timing, these are cracks caused by higher stresses than the strength of the joint in the form of cold cracks. The resulting sound level peaks run into the sound sensors saturation range of 100 dB. In the corresponding graph of the sound energy, a clear gradation can be seen between the background noise, the welding process and the sound event during crack formation (note logarithmic axis scaling). Between the background noise and the welding process lies a factor of approximately 10,000–100,000 eu (1 eu = 1 aJ = 1 × $\:{10}^{-18}$ J) and a factor of approximately 2000–20,000 eu between the welding process and the two signal peaks. For the fault type pore line (cf. Figure 2b), it can be seen that the sound level is reduced by approx. 10 dB and the sound energy by a factor of 1000. For the weld irregularity incident point (cf. Figure 2c), a clear drop in the signal can be seen in both the sound level and the sound energy. The sound level is reduced by 35 dB and the sound energy by a factor of 2300.

**Fig. 3:**
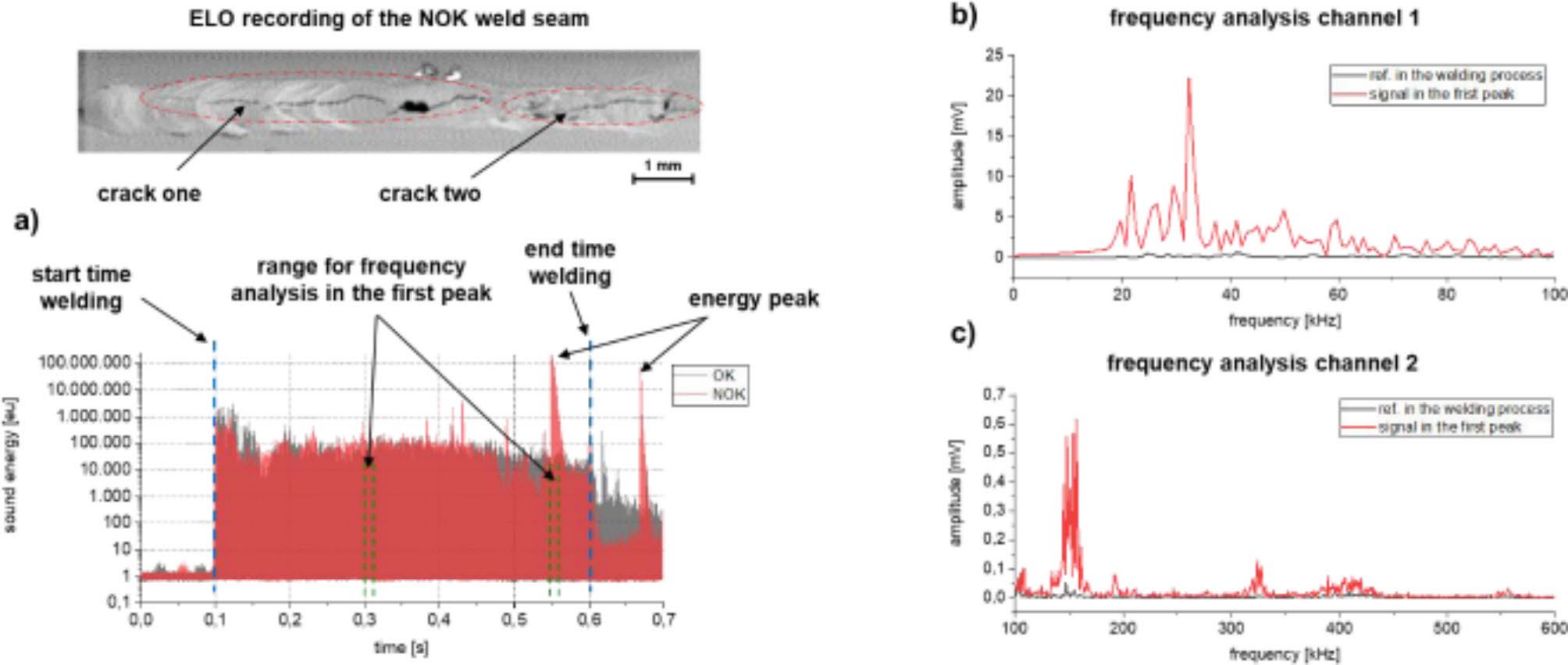
Section by section frequency analysis of a welding process with occurring fault type crack. (**a**) Sound energy curve with the temporal ranges for frequency analysis; (**b**) frequency analysis of the low-frequency sound sensor and (**c**) frequency analysis of the broadband sound sensor.

In order to obtain additional insight on the nature of the respective, defect-induced sound events, a detailed analysis in the frequency and sound energy domain was conducted. Figure 3 shows the fault type crack seen in Fig. 2a including the evaluation of the acoustic energy as well as a frequency analysis. For the frequency analysis, two temporal ranges were separated and compared. Naturally, the length of the separated area depends on the duration of the sound event, in this case $\:{6\times\:10}^{-3}\:s$ during the first peak. For reference during the welding process, an identical signal duration was separated (see dashed green lines in Fig. 3a). In Fig. 3b, the signal for the low-frequency sensor is shown at the two distinct signal ranges. For this purpose, the emitted frequency spectrum of the reference condition (black curve) as well as the sound peak (red curve) were superimposed in a frequency range of 0–100 kHz. It can be seen that more sound is emitted within the first peak in the sound level in the frequency range considered. Particularly in the frequency range between 20 kHz and 40 kHz, a clear increase in the signal can be seen. Figure 3c shows the frequency spectrum from 100 to 600 kHz for the broadband sensor. Analogue, there is also an evident increase in the signal amplitude. The areas at 110 kHz, 150 kHz, 200 kHz, 330 kHz and 420 kHz stand out from the other measurement data and, moreover, the reference weld. As the results indicate, the fault type cracks can be clearly seen in the sound energy as well as in the frequency spectrum using acoustic emission sensing. This is due to the fact that an intensive sound event occurs as a result of the weld seam irregularity, i.e., cold cracking of the weld.

**Fig. 4:**
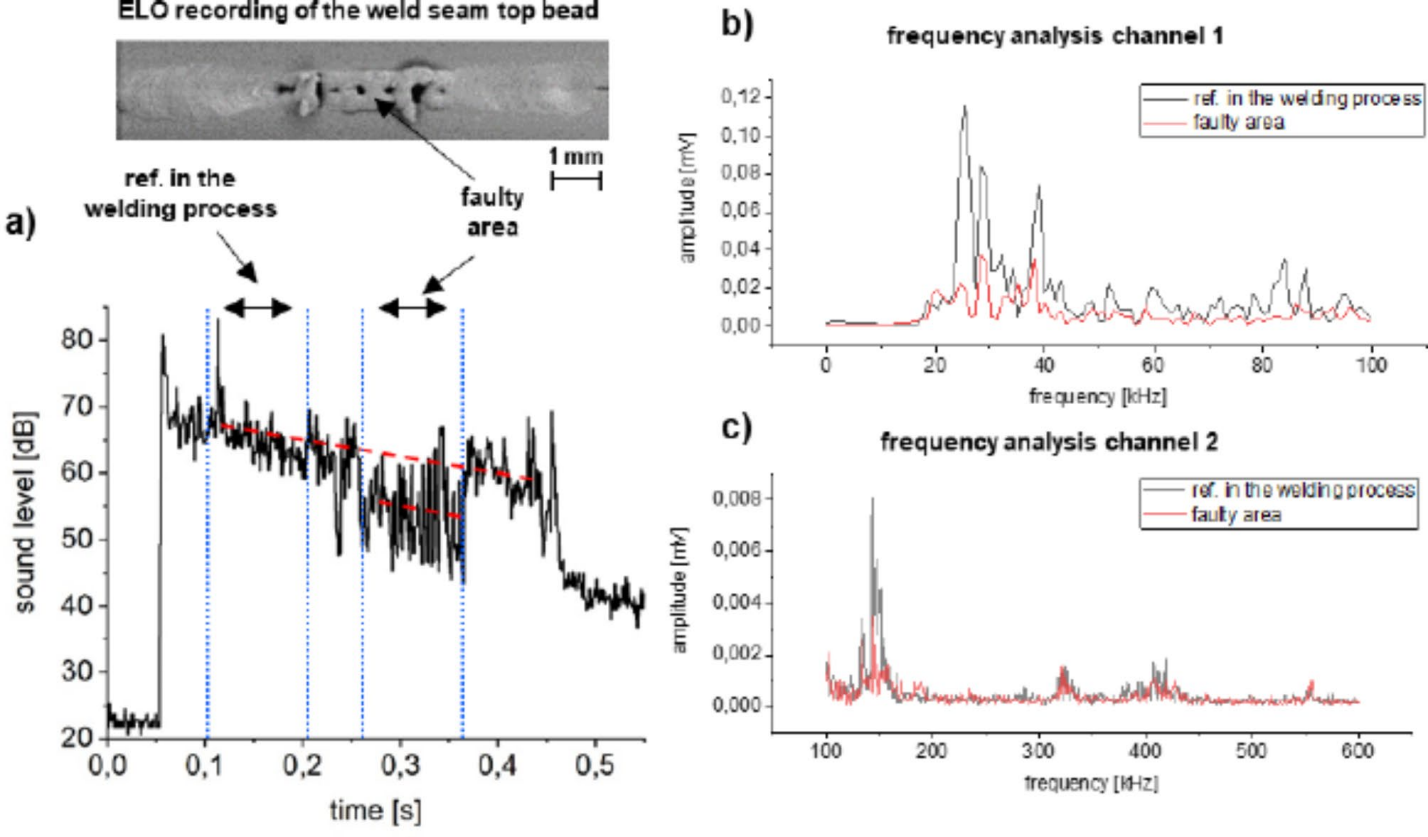
Section-by-section frequency analysis of a welding process with occurring fault type pore line. (**a**) Sound level curve with the ranges for frequency analysis; (**b**) frequency analysis of the low-frequency sound sensor and (**c**) frequency analysis of the broadband sound sensor.

The principle of section-by-section frequency analysis is illustrated in Fig. 4 for the fault type pore line. As before, a defect-free area of the OK weld process was compared with that of the abnormality (see Fig. 4a). Figure 4b, c again show the frequency spectrum from 0 to 100 kHz and from 100 to 600 kHz, respectively. It can be seen that in the range of 20–40 kHz, a higher noise amplitude is detected during the reference welding process. In the frequency range of the broadband sensor, the emitted process noise is also reduced in the range of 150 kHz as well as 420 kHz for the faulty weld in contrast to the reference weld. The other frequency ranges remain similar.

**Fig. 5:**
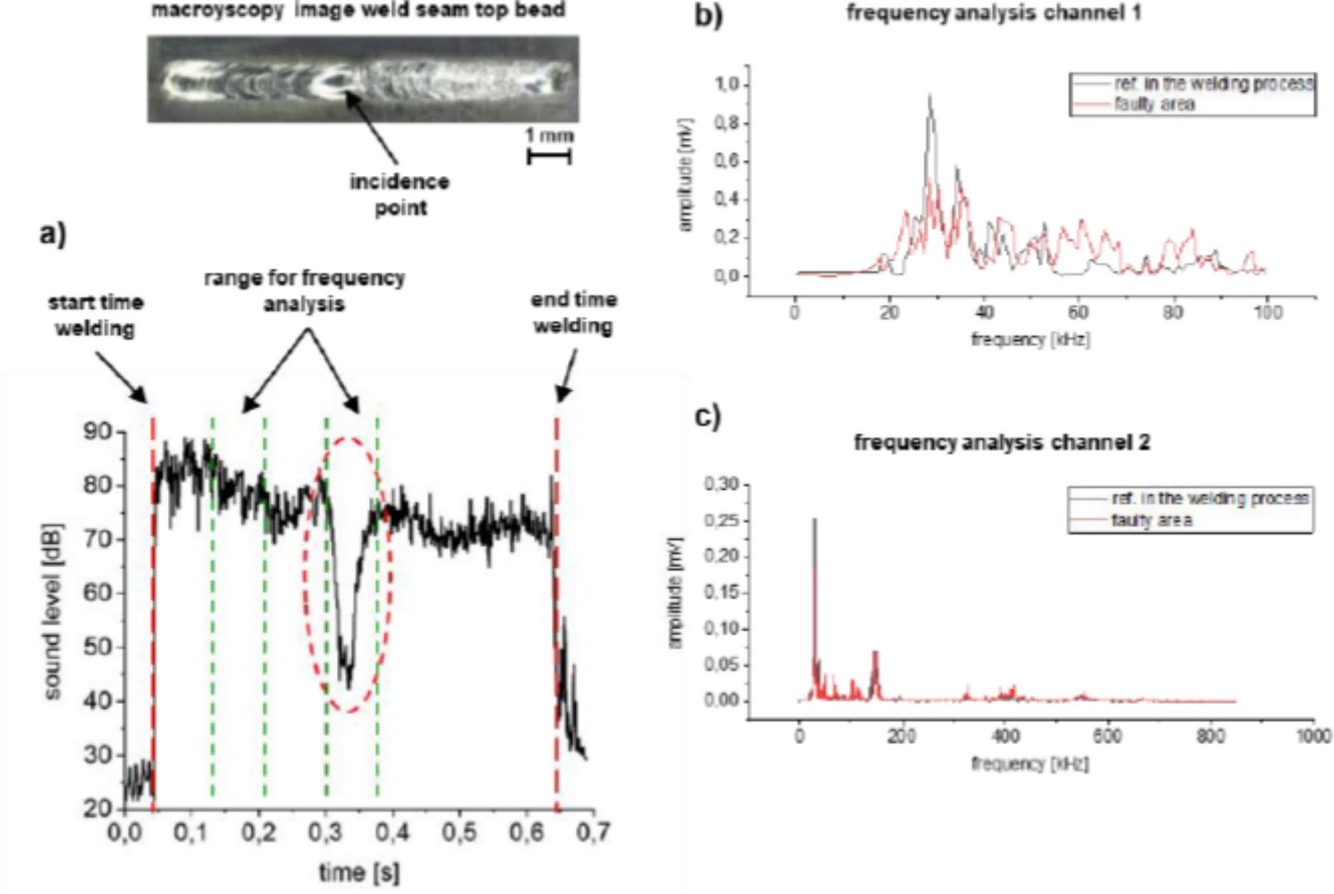
Section-by-section frequency analysis of a welding process with the fault type incident point. (**a**) Sound level curve with the ranges for frequency analysis; (**b**) frequency analysis of the low-frequency sound sensor and (**c**) frequency analysis of the broadband sound sensor.

Figure 5 also shows an analogue analysis for the fault type incidence point. Here it is also noticeable that changes in the emitted frequency spectrum occur mainly in the range 20–40 kHz and at 80–90 kHz. The frequencies in the higher range coincide.

**Fig. 6:**
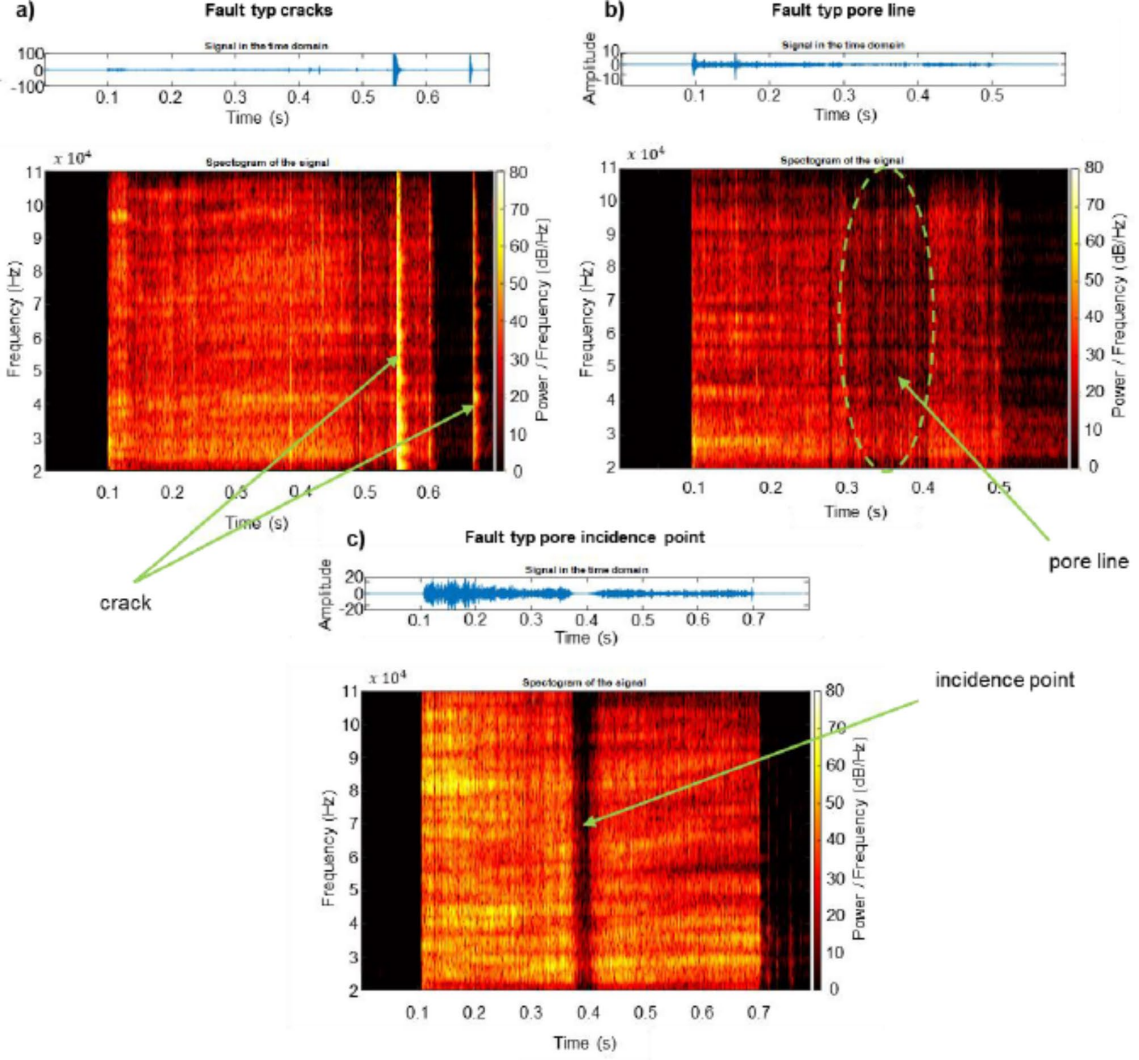
Comparison of the raw sound signal and the STFT for the three fault types (**a**) crack, (**b**) pore line and (**c**) incidence point.

In addition to the frequency analysis of the time signal, an advanced analysis of the sound data can be performed using an adapted Short-Time Fourier Transform (STFT) as well as adapted machine learning models. Figure 6 shows a comparison of the analysis of the measured data using STFT. Here, the time signal (shown as blue line graph atop) is divided into 1 ms sections and a Fourier transformation is performed. Subsequently, the frequency spectrum of the signal with the temporal resolution of 1 ms can be plotted in a graph, where the time (in seconds) is plotted on the x-axis, the frequency band (in kHz) on the y-axis and the amplitude of the frequency spectrum (in dB) on the z-axis, reflecting the change of the emitted frequencies over time. For the fault type crack (cf. Figure 6a), the left spectrogram of the signal shows the frequency of the welding process in the range from 20 to 110 kHz. It can be seen that there is a clear increase in the amplitude of the frequency spectrum at the time of the first and second crack, as was already evident in the time signal. For the fault type pore line (cf. Figure 6b) a slight deviation of the frequencies becomes apparent in the area of the defect, especially from about 40 kHz upward. The spectrogram of the signal for the incident point fault type (cf. Figure 6c) illustrates a clear dip of the emitted frequencies over the entire spectrum.

### Implementation of machine learning models for rapid in-line detection and defect categorization

**Fig. 7:**
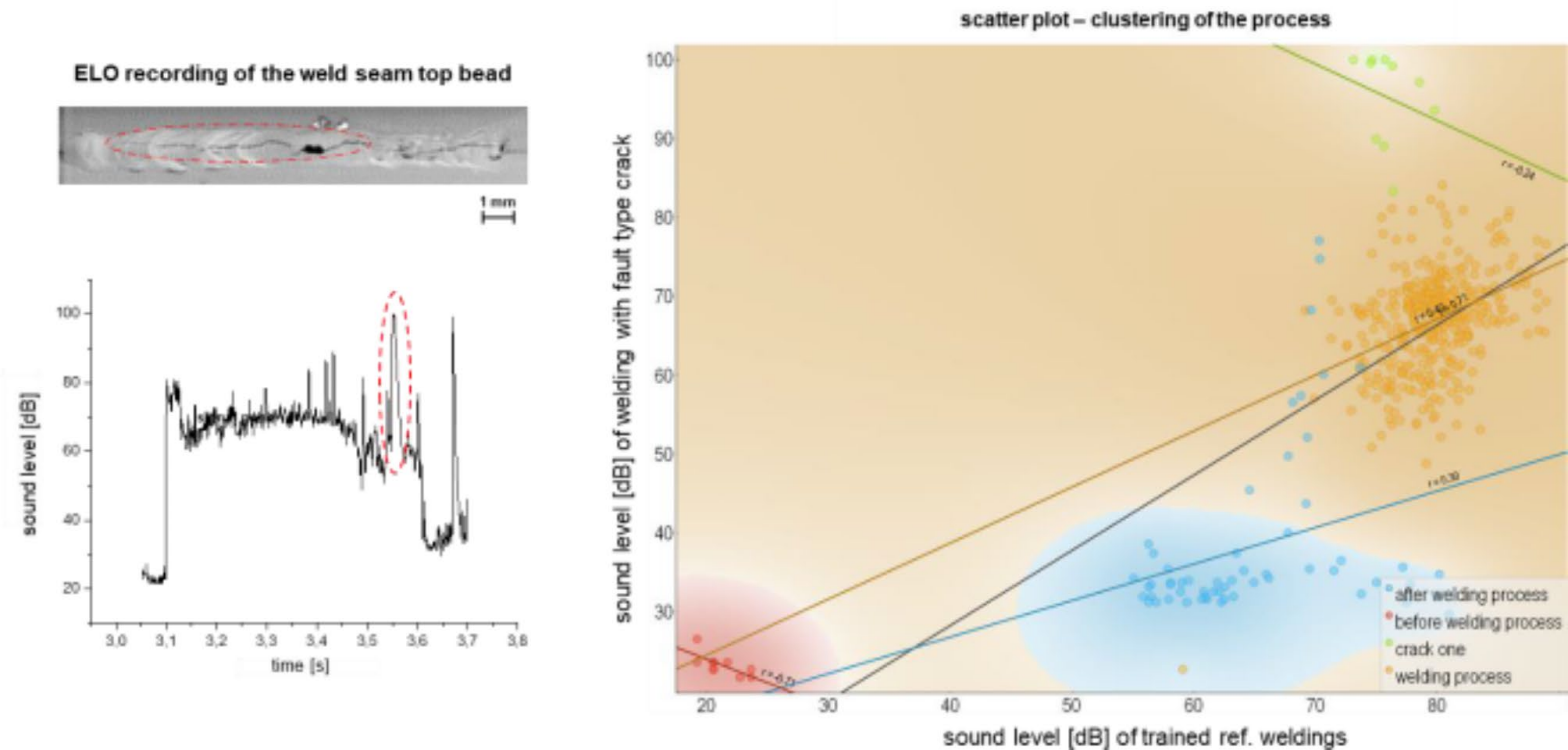
Use of the trained model for clustering of the faulty process using the example of the fault type crack.

**Fig. 8:**
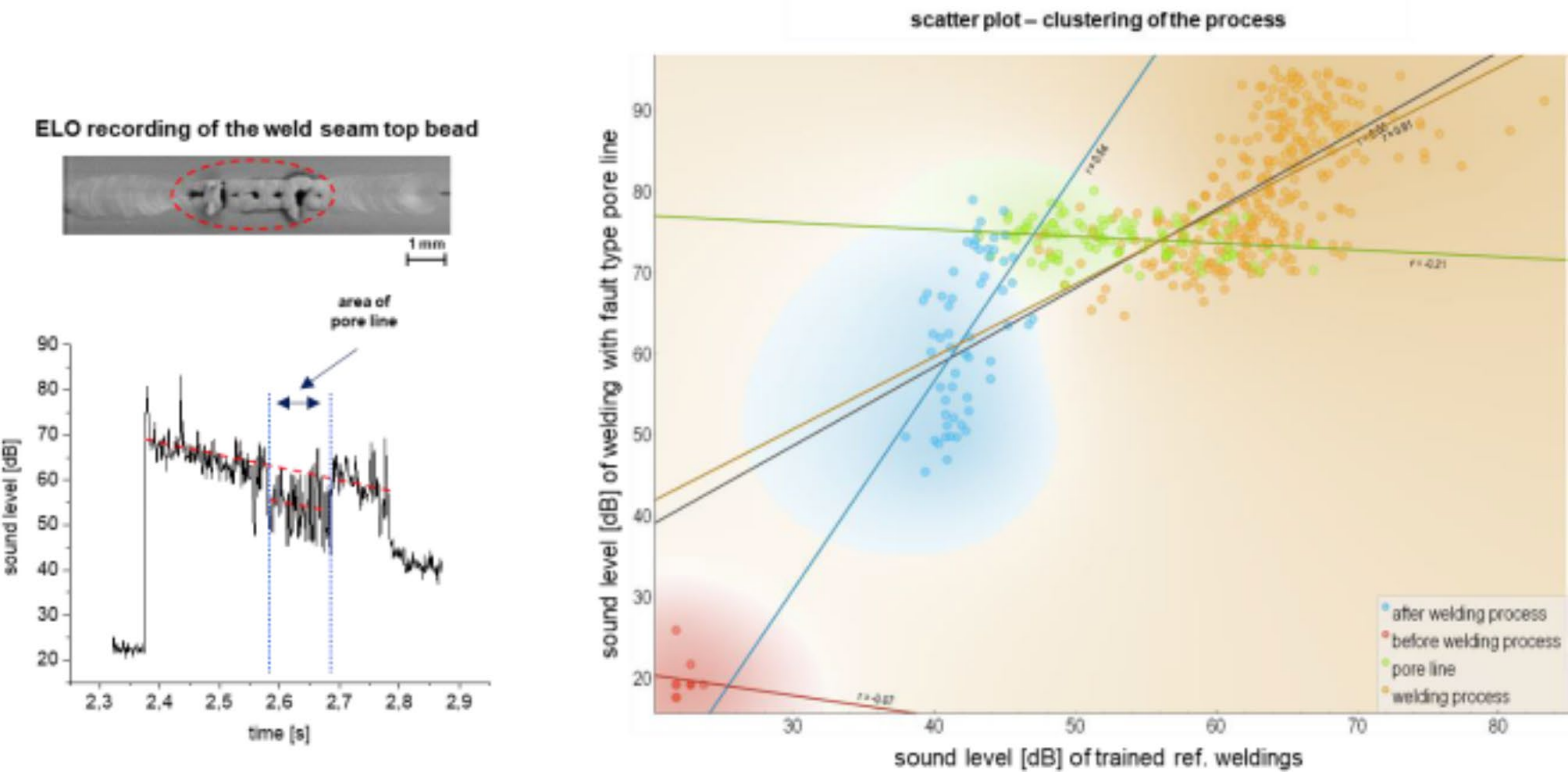
Use of the trained model for clustering of the faulty process using the example of the fault type pore line.

**Fig. 9:**
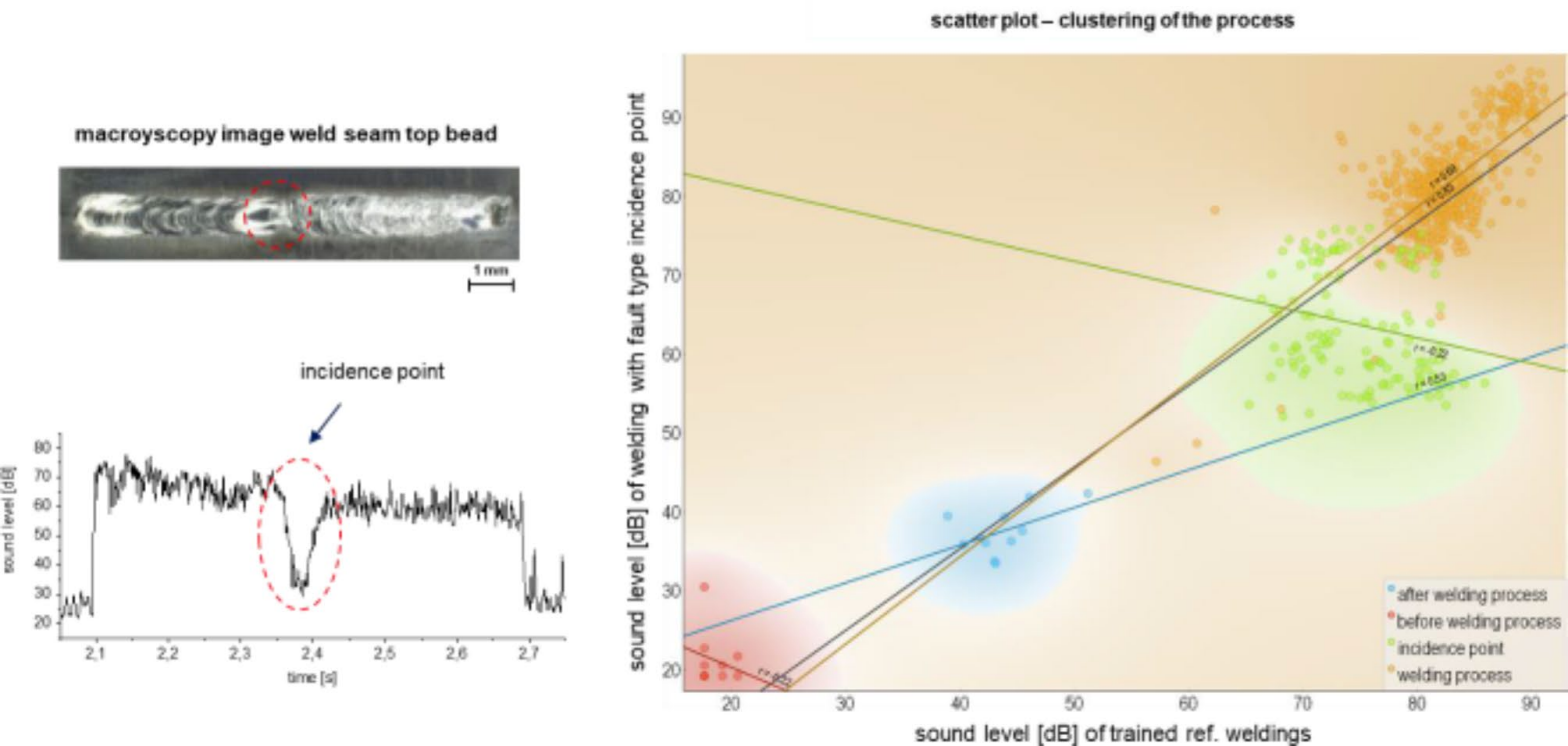
Use of the trained model for clustering of the faulty process using the example of the fault type incidence point.

In addition to the analysis of the sound data in the time and frequency domain, an analysis of the data using pre-trained machine learning (ML) models was performed. In the following Figs. 7, 8 and 9, the trained linear regression model was used to compare an OK weld with a faulty weld as well as to cluster the data. For information on the selection of the model, the reader is referred to the experimental section of this paper. In addition to the ELO recording as well as the sound level presented beforehand, the point cloud diagram with the clustering of the process is shown on the right side. In it, the respective regression lines of the detected areas, i.e., before the welding process (red), after the welding process (blue), the OK welding process (orange) and fault type (green), are calculated and plotted by the model. In the scatter plot, the sound level in dB is shown on the y-axis in each case. In Fig. 7, the sound data of the flaw type crack was examined with the pre-trained model. Here it can be seen that the regression line of the data points from the detected crack shows a very strong deviation (*r* = − 0.24 compared to the reference welding process (*r* = 0.4). Likewise, the model was able to detect the areas before and after the welding process through their differences in the slope r at − 0.21 and 0.30, respectively.

For the fault type pore line, the same evaluations were performed with the pre-trained model and is shown in Fig. 8. It can be seen that the data points of the OK process are very close to those of the trained model. The defective region through the pore line has a significantly different slope of the regression line of *r* = 0.21 instead of the OK Process of *r* = 0.8, as do the regions before and after the welding process. This means that despite the spatial proximity of the points within the diagram, the areas can be clearly distinguished from each other mathematically and can be recognized by means of the ML model.

In Fig. 9, the same analysis is shown for the incident point fault type. Within the point cloud diagram on the right-hand side, a comparison between the trained model and a defective weld is also shown evaluated as sound level. Here it can also be seen that the regression line of the data points in the area of the point of incidence has a slope of *r*=-0.22 instead of the OK process with *r* = 0.83. It is striking that the slope of the regression lines of the fault type pore line as well as that of the fault type incidence point are almost identical and show a similar deviation to the OK process.

## Discussion

### Effects of irregularities on sound signals

As shown in Chap. 3.2, each weld seam irregularity has a distinctive influence on the emitted process noise. In the acquired sound data, it could be shown that the irregularities possess a pronounced influence on both the sound level and the sound energy in the time domain. These results confirm the previous investigations in the field^15,16,32,33^. In addition, it was shown that it is possible to identify various weld seam irregularities by means of specific sound signatures based on the defect-related changes in process emissions. The fault type crack can be recognized by a significant, short-term increase in the sound signals. A reduction in both the sound level and the sound energy occurs for the defect types incidence mark and pore lines. Evidently, the observed differences in sound characteristics stem from the alternating metallurgical nature of the defects. While the cold cracks were caused by embrittlement due to the formation of intermetallic phases, the other two defects are mainly due to evaporation or lack of material within the melt. Obviously, the violent sound signature of the crack in both other defects is not replicated and, thus, indicates that the sound signature of defects is distinctively different depending on their occurrence within the temperature-depended aggregate state.

Furthermore, the occurrence of the aforementioned irregularities could be analyzed in real time, i.e., *in operando*, for the first time. This makes it possible to directly assign the irregularity to a location within the weld seam, except for the defect type crack. Since the defect type crack can only occur with a time delay due to its physical nature, at least three sound sensors are required for the localization measurement. This allows exact localization to be carried out using the sound propagation speed of the material and the distance the sound travels between the sound event and the sound sensor. However, it must be taken into account that small surface cracks that are < 10% of the material thickness can be distorted due to temperature variations and, thus, the position may be displayed incorrectly.

Furthermore, the presented results evidence that the investigated irregularities influence the frequency spectra emitted by the process. Here, a significant increase in the frequencies occurs particularly for the defect type cracks in the frequency ranges of 20–100 kHz; at 150 kHz and at 330 kHz. Obviously, this observation underlines the rationale for both sound level and sound energy presented previously, where the crack formation is apparent due to its violent nature and increases in signal values, which is in good agreement to previous reports in literature with regard to AE^37^.

For the fault types pore line and incidence mark, where the signal is reduced in the time domain, the emitted frequencies are also slightly reduced. By combining the time signals and frequency spectra and by defining threshold values, a reliable detection of weld seam irregularities specific sound signatures is clearly feasible.

Furthermore, it was shown that the ML models used have a low error tolerance of 4.764E-12% in relation to the training data. As the authors confirmed in^38^, an error accuracy of the trained model of > 97% i already sufficient to reliably classify the fault types. With the help of the ML-models it could be shown that the process data can be reliably clustered into the distinctive areas *before the welding process*, *welding process (reference)*, *after the welding process* and *defect*. The regression lines of the individual areas show a significantly different slope for the defects than those within the OK welding process. This results in a high potential as well as a high reliability for automatic defect detection. Again, the differences in the physical nature of the defect formation seem to play a decisive role in their sound signatures, as the slopes of the defect type pore line and incidence point align with reasonable accuracy, thus indicating comparable underlying mechanisms.

## Conclusion

In this publication, the suitability of in-line process monitoring of the EBW process exploiting structure-borne, sound-based acoustic emission was demonstrated using the example of three occurring weld seam irregularities. Furthermore, the general conditions for the acquisition of process noise by means of a structure-borne, sound-based measurement system as well as its implementation at a micro electron beam facility were shown. Furthermore, three fault types and their introduction methods were described, on which the evaluation of the recorded process noises was carried out. The recorded sound data were evaluated in both the time and frequency domains as well as analyzed using pre-trained machine learning models. In summary, the following findings can be derived from the presented results:

- Confirmation of suitability using structure-borne, sound-based AE for *in operando* analysis of EBW.
- The defects can be detected and classified using a combination of different evaluation methods in the time and frequency domain.
- Cracks are characterized by a very strong increase of the sound level, the sound energy as well as the emitted frequencies, which is in good agreement to previous reports on AE in literature and does stem from the violent nature of the physical event. Furthermore, a clear detection through the pre-trained ML-model is possible based on the varying slopes of the clustered data.
- Pore lines can be detected by a deviation of the average value (approx. 10 dB lower) as well as reduction of the sound energy and emitted frequencies. An unambiguous detection of the fault is possible by using the trained ML-model due to the drastically different slope of the regression through the erroneous data cluster.
- A very strong reduction of the sound level is observable for an incidence point. Furthermore, a decrease of the sound energy as well as frequency spectrum is evident. Also, a clear distinction of the fault is possible by using the trained ML-model.
- The used linear regression model for the clustering of the sound data shows a high potential for a clear recognition of process deviations which is confirmed by the calculated regression line (see experimental part).

Finally, it can be stated that a combination of the analyses of the time signal, the frequency spectrum, and the application of ML-methods allow for an unambiguous fault detection. The methods shown here have a very high potential for an in-line integration into existing EBW machines, thus rendering *in operando* analysis and defect detection possible. Furthermore, a reliable detection of cracks is possible, which are inadmissible according to DIN EN ISO 13919-1 in all evaluation groups, which contributes to a significant improvement of the quality control, especially of critical welds. In addition to that, the high potential of an evaluation by means of STFT and the use of ML models as a basis for evaluation could be shown, which enables an automated quality assessment in the process during every weldment, effectively facilitating a 100% quality assessment without additional process times downstream.

Future investigations need to build on the presented means of identification and expand the portfolio of detectable weld seam irregularities, for example inappropriate component positioning, humping or insufficient penetration. Clearly, an in-depth evaluation of their defect-specific sound signatures is necessary, but the investigation at hand evidently underlines the effectiveness of the approach utilizing AE monitoring in combination with ML-models during EBW.

## Experimental procedure and materials

### Welding procedure and AE detection

In order to record the process noise during EBW, a number of general conditions must be taken into account. To reduce sound losses, there should be as little planes of separation as possible between the joining partners and the structure-borne sound sensor. Since the frequency range of the sound emitted during the welding process was not yet known at the beginning of the experimental tests, the broadest possible frequency spectrum should be covered. Furthermore, the size of the sensor must be taken into account, since the vacuum chambers of electron beam systems are designed to be only as large as is required for the welding task. This avoids long evacuation times. The following experimental configuration results from the above-mentioned framework conditions and the system technologies used.

A micro electron beam system (SEM 108, pro-beam, Germany) was used for the welding experiments. The EBW system was supplemented by a set of two acoustic emission sensors to record the structure-borne process noise. The first sensor was a low frequency sensor that features a high sensitivity in the frequency range of 20 kHz to 85 kHz (Vallen Systems VS30-V, Vallen Systeme, Germany). The other sensor was a broadband sensor that covers the frequency range from 50 kHz to 1 MHz (Vallen Systems Vs900-M, Vallen Systeme, Germany). For datasheets of the respective sensors, the reader is referred to^39,40^. Through the use of these two sensors, it was possible to detect and analyze a wide frequency spectrum during the welding process. To avoid signal loss due to material transitions, both structure-borne sound sensors were mounted directly on the clamping device, therefore fulfilling the general considerations mentioned beforehand.

Since the fault-specific sound signatures were to be identified here, continuous recording and evaluation of these measurement data was considered most senseful. From the recorded measurement data, the sound level, the linear amplitude, the sound energy as well as the frequency spectrum were evaluated. The sound energy was calculated according to DIN EN 1339-9 as an integral of the squared sound signal.

**Fig. 10:**
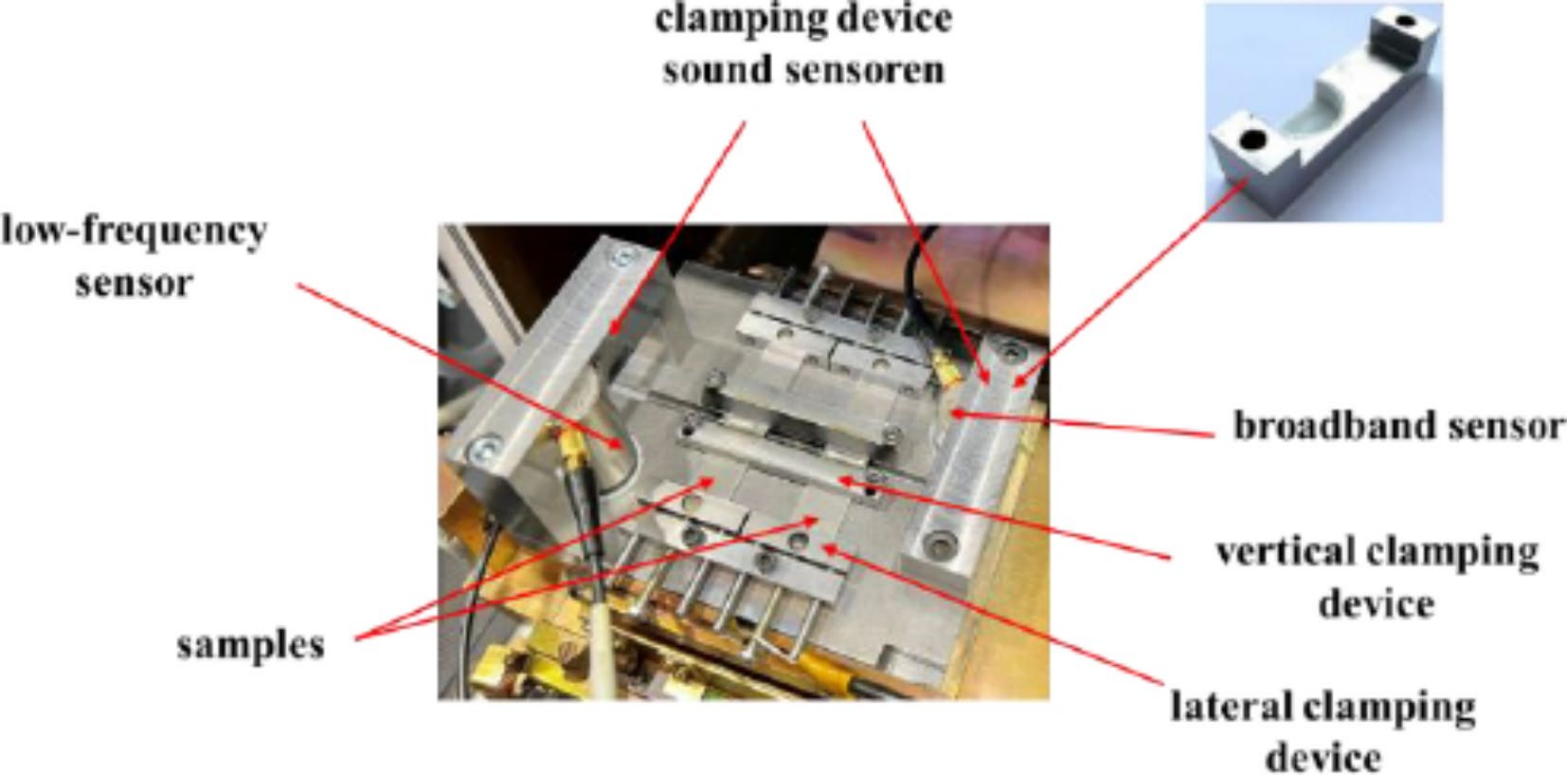
Overview of the clamping device in the electron beam chamber with mounted sound sensors.

Figure 10 shows the clamping device within the electron beam system. Two welds could be performed immediately after another. The employed sound sensors directly attached to the clamping device by clamping bars and vacuum grease with a resistance of up to $\:{10}^{-6}\:mbar$ as the contact medium. Due to the direct positioning on the clamping device, only one transition surface was available. Circular recesses have been milled in the clamping bars in order to obtain a constant position and thus the best possible reproducibility of the sensor positioning on the clamping device. The contact pressure between the sound sensor and the fixture can be adjusted via the tightening torque of the screws.

 The planar specimen dimensions employed for all tests were 25 mm × 12.5 mm. The materials used were an austenitic stainless steel (AISI304) with a thickness of 1 mm, an aluminum alloy (AlMg3) and a magnesium die-casting alloy (AZ91), the latter both with a thickness of 1.5 mm. The welded joints with the stainless steel and the aluminum alloy were welded together in the same way as well as an aluminum-magnesium mixed joint. The specimens were joined along the short edge to form similar (stainless steel, aluminum alloy) and dissimilar (aluminum to magnesium alloy) joints. To obtain the weld edge preparation necessary for electron steel welding, the specimens were extracted from sheet material along the joint edge using electric discharge machining to provide a zero-gap when clamped. An accelerating voltage of 60 kV was set for all welding tests. The welds with the stainless steel were performed at a welding speed of 17 mm/s and a beam current of 4.4 mA, and the similar aluminum and dissimilar aluminum-magnesium welds at 20 mm/s and 7 mA.

### ML model development

**Fig. 11:**
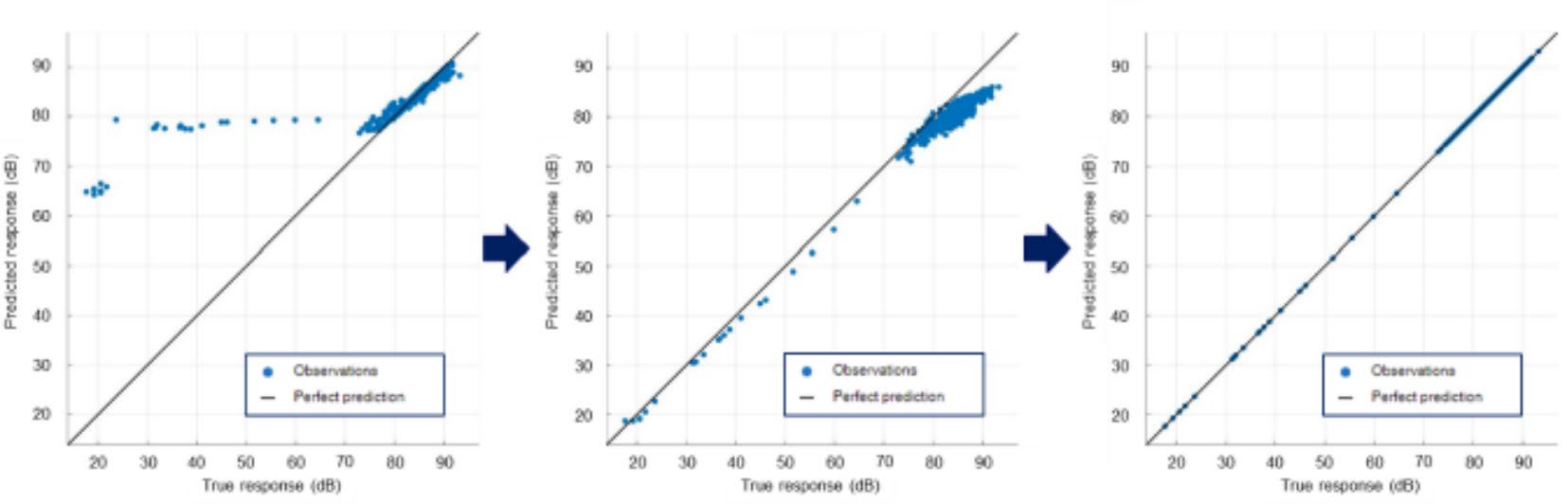
Comparison of different trained machine learning prediction models and their accuracy of the trained model.

For the development of the ML-models, the measurement data had to be converted into a neutral file format. Furthermore, to be able to fully analyze the data with the set sampling rate, the raw data needed to be exported. The extracted time signals were then ready for the model-based evaluation. The data obtained from 100 reference welds of AISI304 was used to test the suitability of different ML-models. Different approaches and parameter variations were tested and optimized iteratively. Figure 11 shows the results for three different models. The response predicted by the model is plotted on the y-axis while the actual response is plotted on the x-axis, resulting in an ideal match on the diagonal. A fine-meshed Gaussian support vector machine was used in Fig. 11a. Analyzing the data with this model showed a comparatively good agreement during the welding process, but not at the beginning and end of the welding process. The training time for the data set used was 9.76 s and the root mean square error between the different arrays (root means square error, RMSE) was 8.29%. In Fig. 11b, a reinforced tree structure was used as a statistical learning method for analysis of the reference data. Evidently, the trained model was able to capture the start and end areas much better, but the error increased slightly during the welding process. The model required a training time of 12.53 s with an RMSE value of 3.8%. In Fig. 11c, the linear regression approach was used to train the model. It can be seen that the response of the trained model is almost ideal with respect to the above specifications along the diagonal. Compared to previous models, the RMSE value was only 4.764E-12%, which was significantly lower than the others. Further values of the trained linear regression model are: MSE (mean squared error) 2.2695E−23; MAE (mean absolute error) 3.6474E−12 and R-Squared 1.00. However, the training time was only 9.80 s despite the significantly lower error. A linear interaction was used as the default setting for the model.

**Fig. 12:**
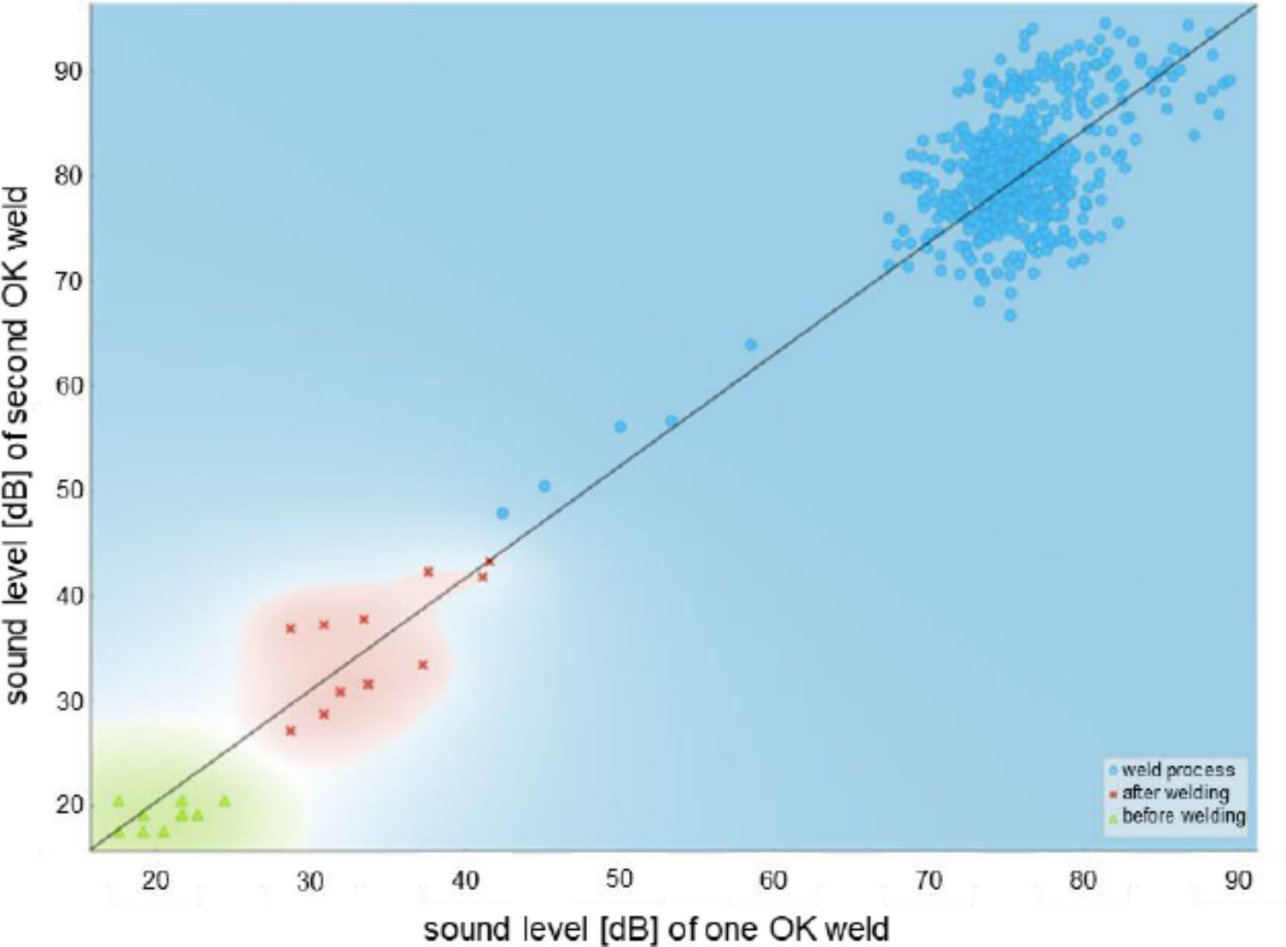
Clustering of the sound level for channel 1 in dB before, during and after the welding process using gradient boosting.

 The linear regression model was thus selected from the results of the training models for the subsequent investigations in this paper. In addition, the so-called gradient boosting method was used as a further calculation model to determine and minimize errors and to classify the sound level of channel 1 into the classes before, during and after the welding process. The 100 data sets from the reference welding tests with AISI304 were also used to create and train the model. The following parameters were set as the basis for the gradient boosting model: number of trees: 100; learning rate: 0.100 and reproducible training. As extended parameters, the limit of the depth of the individual trees was restricted to three and it was specified that subsets smaller than two were not divided. Furthermore, the proportion of the training instance was set to 1.00 for the sub-sample. The trained gradient boosting method was then used to compare the data from two welds with the trained model. The comparison is shown in Fig. 12. The model was able to automatically recognize the data during the welding process (blue dots), after the welding process (red crosses) and before the welding process (green triangles) and classify them into the corresponding groups.

## Data Availability

The datasets used and/or analysed during the current study available from the corresponding author on reasonable request.
